# Effect of ZFN-edited myostatin loss-of-function mutation on gut microbiota in Meishan pigs

**DOI:** 10.1371/journal.pone.0210619

**Published:** 2019-01-15

**Authors:** Wen-Tao Cui, Gao-Jun Xiao, Sheng-Wang Jiang, Li-Li Qian, Chun-Bo Cai, Biao Li, Shan-Shan Xie, Ting Gao, Kui Li

**Affiliations:** 1 Institute of Animal Sciences, Chinese Academy of Agricultural Sciences, Beijing, P R China; 2 State Key Laboratory of Agrobiotechnology, China Agricultural University, Beijing, P R China; University of Illinois, UNITED STATES

## Abstract

Intestine contains the body's second largest genetic information, so a relatively stable microbiota ecosystems and interactions between intestinal micro-organisms play a pivotal role in the normal growth and development in animals. The establishment of intestinal microflora is affected by a variety of factors such as species, environmental factors, developmental stage, organizational structure and physiological characteristics of various parts of the digestive tract. Gene editing technology such as ZFN has recently been used as a new approach to replace the traditional transgenic technology and to make genetic modifications in animals. However, it is not known if genetic modification by gene editing technology will have any impact on gut microbiota. In this study, by sequencing 16S rRNA collected from rectum, we investigated the effects of ZFN-mediated myostatin (MSTN) loss-of-function mutation (*MSTN*^*-/-*^*)* on gut microbiota in Meishan pigs. Our results showed that the fecal microbial composition is very similar between *MSTN*^*-/-*^ Meishan pigs and wild type Meishan pigs. Although significant differences in certain individual strains were observed, all the dominant microorganism species are basically the same between *MSTN*^*-/-*^ and wild type pigs. However, these differences do not adversely affect *MSTN*^*-/-*^ Meishan pigs. Thus, it is concluded that ZFN-mediated MSTN loss-of-function mutation did not have any adverse effect on the gut microbiota in Meishan pigs.

## Introduction

With recent progress in the improvement of pig breeding system in China, pigs with good feed efficiency and high reproductivity rate are becoming more popular and are thus introduced in the livestock industry. Meishan pigs are a locally famous breed in China, and are well known for their high prolificacy and early sexual maturity. For example, Meishan sows can have an average birth of 16 piglets per sow and as high as 33 piglets per sow. However, this breed has a high percentage of carcass fat and poor feed efficiency[1].

Myostatin (MSTN) is a negative regulator of skeletal muscle growth and development. It has been established that naturally occurring loss-of-function mutations in *MSTN* gene can result in a significant increase in skeletal muscle mass with a simultaneous reduction in body fat in cattle[2], sheep[3], dogs[4], and humans[5]. Due to the specific function of MSTN in skeletal muscle growth and development, MSTN gene becomes an important target for gene editing technology in livestock animals to improve meat quality with higher percentage of lean yield and lower percentage of fat.

To fully utilize the advantages of Meishan pigs as described above, the meat quality from Meishan pigs needs to be improved. Along this line, our lab has recently initiated new studies on porcine MSTN functions in Meishan pigs using gene editing technology, with a goal to increase the lean meat and decrease the fat. In 2015, we have successfully produced MSTN loss-of-function mutant (*MSTN*^*-/-*^) Meishan boars[6]. These *MSTN*^*-/-*^ Meishan boars showed apparent double muscle phenotype with greater lean meat yield and lower body fat. It is expected that *MSTN*^*-/-*^ Meishan pigs could be used to produce high quality pork to meet market’s demand.

From a food safety point of view, currently it is not very clear if genetic modification of endogenous MSTN gene could have any unknown effect on the safety of pork produced by *MSTN*^*-/-*^ Meishan pigs. Our lab recently conducted a subchronic study in rats to assess the safety of pork produced from *MSTN*^*-/-*^ Meishan pigs[7]. The 90-day feeding study clearly indicated that feeding the ZFN-mediated *MSTN*^*-/-*^ pork did not have any adverse effect on the health of rats. Additionally, results from the off-target analysis, blood physiological and biochemical tests, and analysis of nutrient composition in pork showed that no difference was observed between the *MSTN*^*-/-*^ pigs and the wild type pigs[6].

A stable and balanced gut microbiota plays an important role in the physiological and metabolic activities for human and animal health, and therefore the intestinal microbiota represents a key area to study the unknown or unintended effects in genetically modified animals such as *MSTN*^*-/-*^ pigs. The dynamic balance of intestinal microbes is a sign of pig's intestinal health and the basis of healthy growth. Many studies have shown that the composition of dominant microbes in the intestinal floral can significantly affect the health of pigs; Higher content of beneficial bacteria means healthier pigs. We had previously demonstrated this point of view by analysis of microbial sequencing of the intestine from the large white pigs expressing *NEO* gene[8]. To our knowledge, there are few studies focusing on the gut microbiota to evaluate safety issues for livestock animals containing ZFN mediated genetic modifications. In this study, 16S rRNA gene sequencing was performed by high-throughput sequencing technology for fecal samples collected from MSTN loss-of-function mutant (*MSTN*^*-/-*^) Meishan pigs and wild type pigs to detect if there is any difference in gut microbiota under the same feeding conditions. Data from this study will provide very useful scientific information to support the regulatory approval process of the *MSTN*^*-/-*^ pigs as a livestock to commercially produce pork.

## Material and methods

### Ethics statement

The animal protocols contained in this study were approved by the Institutional Animal Care and Use Committee (IACUC) of the Chinese Academy of Agricultural Sciences prior to initiation of the experiment. Care of all vertebrate animals is subject to regular review by the IACUC and complies with Animal Welfare laws and regulations of China. Periodic health evaluations are made by Veterinary Service to ensure that all pigs are healthy, receive adequate housing, feed, and access to water. Daily observations are made to ensure that appropriate standards of animal care are being met.

### Sample collection

Meishan pigs were produced using the same method as previously described[6]. Piglets were maintained in Qingdao animal facility, weaned one month after birth, and then randomly assigned into different pens (each pen hosted 3 pigs so that all animals have enough space to rest and sleep). The environmental conditions of each pen were: temperature 18–24°C, and humanity at 55–65%. These conditions make all animals live in a comfortable environment. Feeding was restrained in the early stage, but the restrain was stopped once the body weight reached to 50kg (please refer to S1 Table for diet information). The drinking water for all experimental pigs was autoclaved prior to feeding so that all animals have healthy food and water. When body weight reached to 90 kg, 4 *MSTN*^*-/-*^ pigs (genetically engineered pigs or GE pigs). and 4 wild type (WT, also refer to *MSTN*^*+/+*^) pigs were euthanized by method of electrocution which was approved by the IACUC, and fresh rectal feces were collected from 4 WT female pigs and 4 female MSTN^-/-^ pigs and placed in liquid nitrogen, then transferred to a -80°C freezer until use. Following sample collection, all animals were humanely euthanized per approved protocols.

### DNA extraction, purification, and 16S rRNA amplification

DNA was extracted and purified from each fecal sample (0.2g) using the Stool DNA Kit (OMEGA, USA) per the manufacturer’s instructions with the following slight modifications: each sample was incubated and mixed with glass bead X for 2 minutes instead of 1 minute. Sterile zirconia beads were added to each sample. For each sample, DNA was extracted in duplicate to avoid bias, and the extracts from the same sample were pooled as one sample. The DNA purity and concentration were analyzed spectrophotometrically using an E-Spect ES-2 (Malcom, Japan). The extracted DNA was stored at -20C until use.

V3-V4 region (468bp) of 16S rRNA amplification was performed using the following primers: Forward Primer: 5'-ACTCCTACGGGAGGCAGCAG-3'; Reverse Primer: 5'-GGACTACHVGGGTWTCTAAT-3'. PCR was carried out, in triplicates, in 50 μl reactions containing 20 μM primer, 30 ng of template DNA, 10 mM dNTPs, 10 x Pyobest Buffer, and 0.75 U Pyrobest DNA Polymerase (Takara Code: DR005A, Japan). The following amplification program was used: an initial denaturation step at 95°C for 5 min, followed by 25 cycles of 95°C for 30s (denaturation), 56°C for 30s (annealing) and 72°C for 40s (extension), and a final extension step at 72°C for 10min. Negative control assays were also performed. For each sample, the PCR products of the triplicates were combined and detected by 2% agarose gel electrophoresis. The PCR product was recovered using the AxyPrepDNA gel recovery kit (AXYGEN), eluted with Tris-HCl, followed by electrophoresis using 2% agarose gel.

### Sequencing and data analysis

The concentration of PCR products was determined with QuantiFluor-ST Blue double-stranded DNA assay (Promega, USA). Sequencing was performed using the Illumina Miseq platform.

Pyrosequencing reads with more than one ambiguous nucleotide or within correct barcodes or primers were removed and excluded from further analysis. Since the Miseq sequence data contain double-ended sequences, the fastq data were first filtered for the poor/low-quality sequences, which were defined as those with an average quality value of <20 over a 50-bp sliding window, were discarded. Sets of sequences with >97% identity are defined as an Operational Taxonomic Unit (OTU) (Usearch,[9]). OTUs are assigned to a taxonomy using the Ribosomal Database Project (RDP) Naive Bayes classifier (Release119 http://www.arb-silva.de). Representative sequences from each cluster were aligned with the PyNAST aligner to the Greengenes (CCBY-SA 3.0) core set in QIIME. A phylogeny was constructed within QIIME using FastTree. Rarefaction curves (which determines the species abundance in samples and if the sequencing data of are reasonable) and alpha diversity which estimates the species abundance and diversity of bacterial communities) calculations were also performed using QIIME [10]. The value of Chao1 is used to estimate the abundance (OTU number) of bacteria in test samples. The value from the observed species is used to detect the actual measured OTU number. As the number of sequences increases, the actually observed OTU number keeps changing continuously. The bigger the observed species D value is, the more of the actually observed OTU number. Phylogenetic diversity (PD) and Shannon diversity index (SI) were estimated to evaluate the ecological diversity of microbiota from each sample. SI is a quantitative measure that reflects how many different types (such as species) there are in a dataset, and simultaneously takes into account how evenly the basic entities (such as individuals) are distributed among those types. The value of a diversity index increases both when the number of types increases and when evenness increases. But the interpretation is hindered by uncertain species definitions and the lack of a statistical framework for comparing values. In contrast to SI, phylogenetic diversity (PD) takes into account the taxonomic breadth of samples without relying on morphotaxa, species or sequence-type designations. To analyze the relationships between samples, dual hierarchal dendrograms were calculated, based on bacterial composition information at taxonomic levels. These analyses were used to bin 16S rRNA V3 gene sequences into OTUs and to display microbial genera partitioning across microbe GI tracts. A spring-embedded algorithm was used to cluster the OTUs and samples[9]. LDA (linear discriminant analysis) Effect Size is used to find species with significant differences in abundance between multiple groups and also subgroups within a group[11]. Statistical analysis Changes in bacterial abundance were compared using repeated measures ANOVA analysis with the Tukey’s honestly significant difference (HSD) post hoc test. Relationships between sequences and diversity and coverage were examined by Pearson’s correlation. Statistical analyses were performed using Graohoad prism Program (version5.0.1, Graphpad software Inc., San Diego (CA, USA). Significance was accepted at P<0.05[12].

## Results

### Microbe distributions in feces MSTN^-/-^ pigs

**Fig 1:**
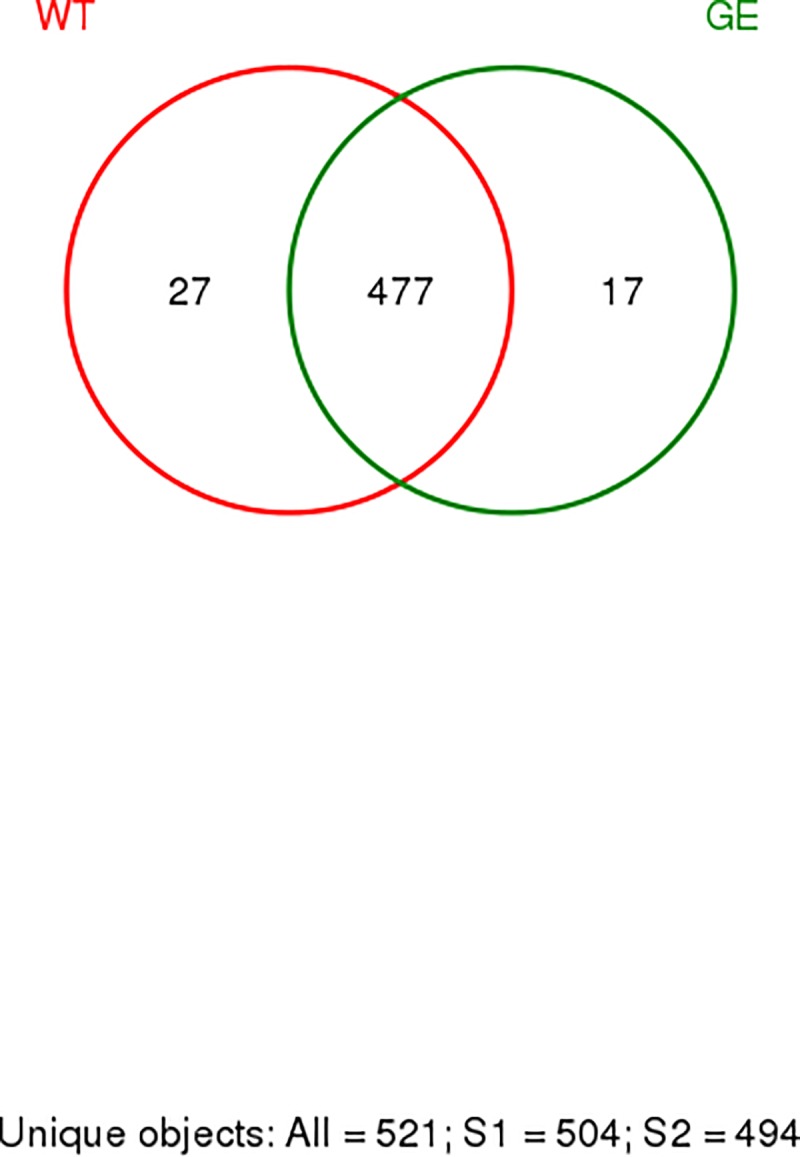
The Venn diagram of the shared and unique OTUs between GE and WT. GE: Fecal samples collected from genetically engineered pigs. WT: Fecal samples collected from wild type pigs.

To investigate the change in intestinal flora in genetically engineered (*MSTN*^*-/-*^*)* Meishan pigs, feces were collected from 4 wild type female pigs and 4 female *MSTN*^*-/-*^ pigs, respectively. Sequencing of 16S rRNA V3-V4 regions was performed, with results being presented in **Table 1**. The reads for each sample is in the range of 23542 to 34399. After quality trimming and chimera checking, each sample has 18722±2004 tags with a minimum length of 360 nucleotides, a maximum length of 480. After operations of classification and RDP Classifier, a total of 521 OTU were obtained. As shown in **Table 1**, OTU distribution in each sample falls in between 303 and 420, including 14 phyla, 22 classes, 28 order, 46 families, 111 genera and 29 species. From the rarefaction analysis (**S1 Fig**), we confirmed that the raw data of sequencing has a high degree of the required coverage[13]. Based on the calculated Shannon index for the raw data from each sample (**S2 Fig**), it is confirmed that the high degree of sequencing consistence was achieved. From the species accumulation curves as shown in **S3 Fig**, it is clear that the number of samples is enough to satisfy this study[14]. These data revealed a complex bacterial community structure and a wide range of diversity in feces from Meishan pigs. As seen in the Specaccum from the Venn figure (**Fig 1**), both wild type and *MSTN*^*-/-*^ Meishan pigs share 477 OTUs, which is 91.6% of total (521) OTUs. *MSTN*^*-/-*^ pigs have 17 unique OTUs, accounting for 3.2% of the total OTUs, while WT pigs have 27 unique OTUs, accounting for 5.2% of the total OTUs. From the above results, it is concluded that the overall microbial distribution is similar between *MSTN*^*-/-*^ group and the WT group, although there are some differences in certain strains.

**Table 1:** Overview of pyrosequencing results of GE and WT.

Sample ID	Raw tags	Clean tags	Final tags	OTUs	Goods coverage
**GE1**	24330	21373	17359	372	0.9948143
**GE2**	28745	25266	20075	375	0.9947116
**GE3**	30288	25756	19459	303	0.9959294
**GE4**	23542	20104	16239	375	0.9942669
**WT1**	30690	25274	19886	386	0.9945064
**WT2**	26054	22198	18002	375	0.9945474
**WT3**	34399	29217	22461	420	0.995471
**WT4**	26122	22106	16295	399	0.9949716

GE: Fecal samples collected from genetically engineered pigs. WT: Fecal samples collected from wild type pigs.

### α-diversity index in MSTN^-/-^ Meishan pigs

**Fig 2:**
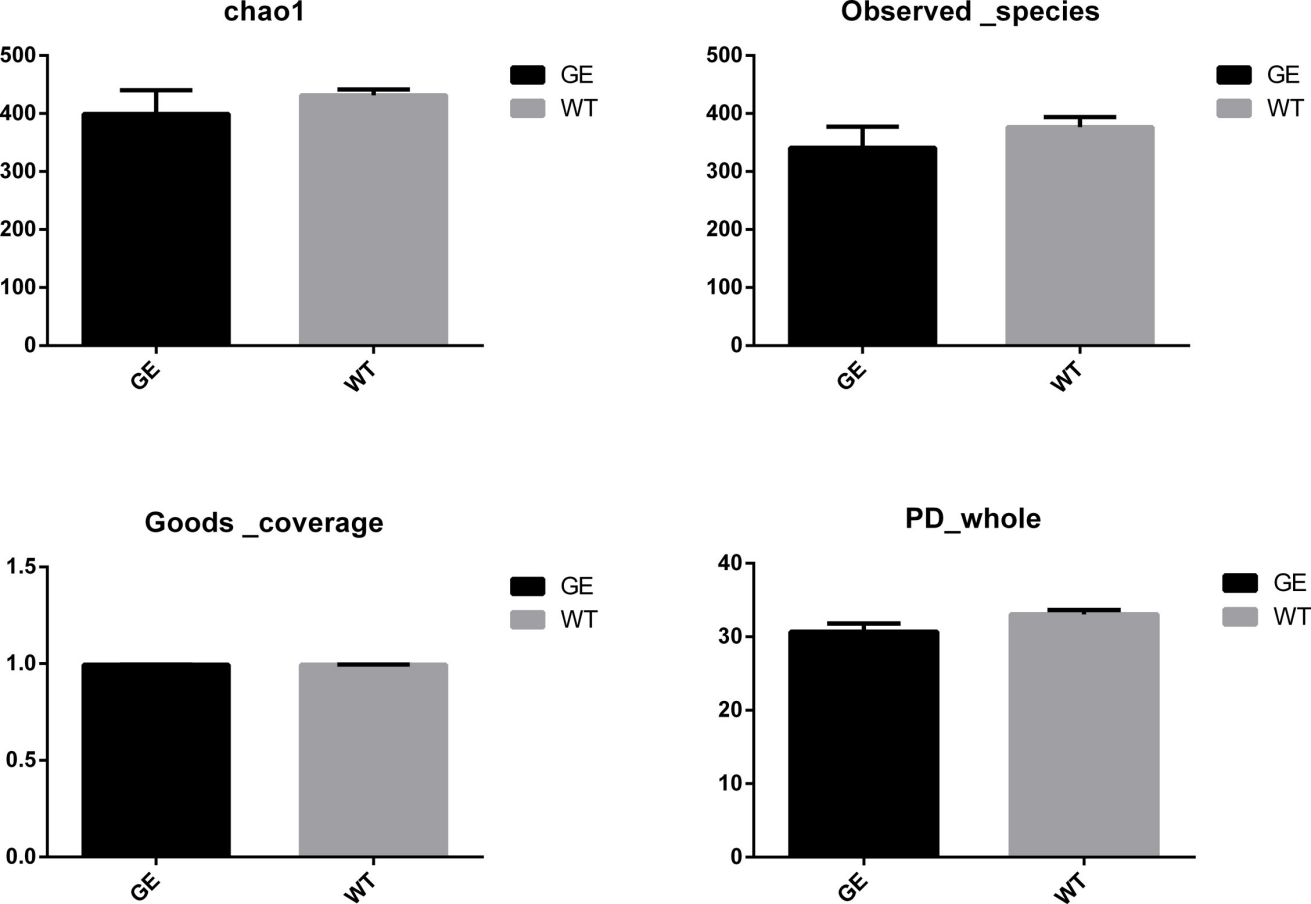
Alpha-diversity comparison of the microbiomes from WT and *MSTN*^*-/-*^ pigs. GE: Fecal samples collected from genetically engineered pigs. WT: Fecal samples collected from wild type pigs.

α-diversity index (**S2 Table**) is used to measure the difference in microbial diversity between different samples. Goods-coverage represents the sequencing coverage for each sample. **Table 1** is the summary of sequencing coverage results for all samples tested. It is clear that the sequencing coverage is above 99% for all samples, indicating that the sequencing efficiency is sufficiently high to detect the sequence in each sample. The Shannon index curve started a linear upward trend at the very beginning due to the lack of coverage, but the curve became flat when the sequencing coverage is sufficient to cover the most microbes (see **S2 Fig**). Analysis of Chao1 and phylogenetic diversity (PD) indicator whole tree showed that higher diversity in WT samples was observed when compared to *MSTN*^*-/-*^ samples. It can be seen from the alpha-bar chat in **Fig 2** that the microbial diversity in the *MSTN*^*-/-*^ group decreases a little bit compared to WT group, but the decrease is not significant **(S2 Table)**.

### Composition of fecal microorganisms and analysis of their differences

**Fig 3:**
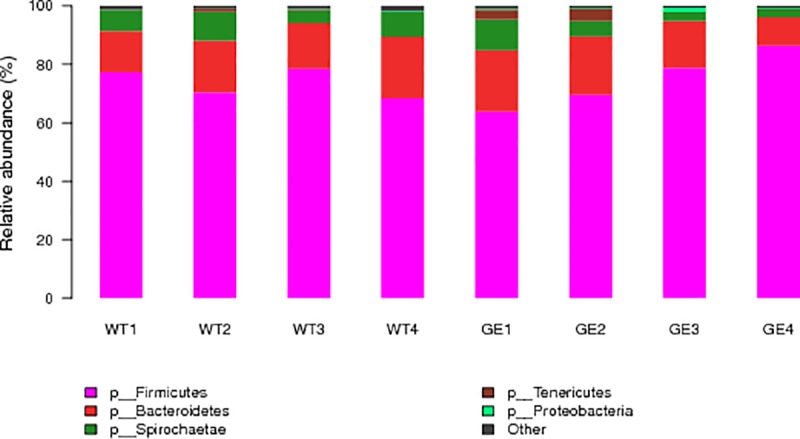
Microbial community structure of fecal samples at phylum level. GE: Fecal samples collected from genetically engineered pigs. WT: Fecal samples collected from wild type pigs. Axis Y is relative abundance of total microbiota.

**Fig 4:**
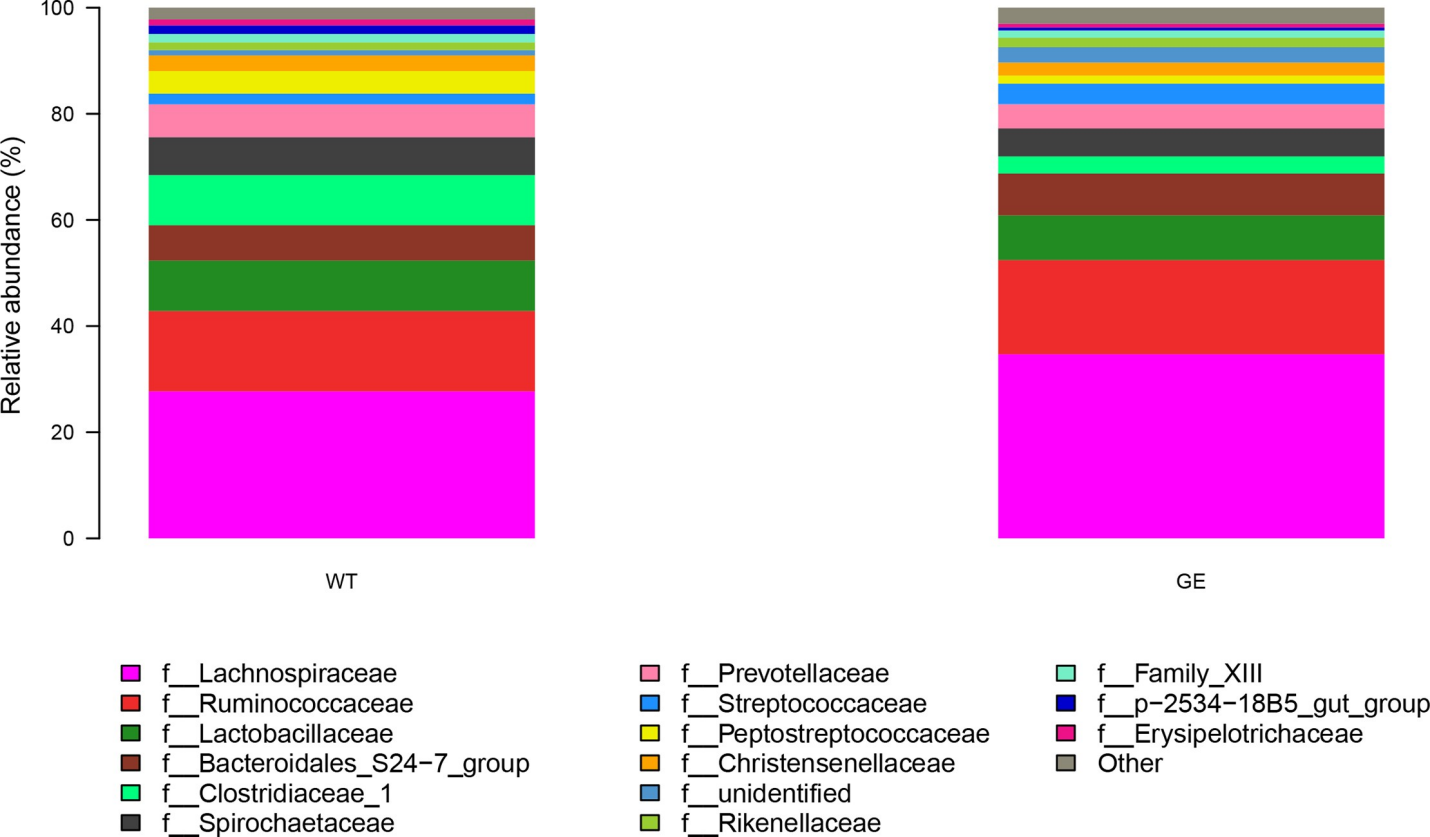
Microbial community structure of fecal samples at family level. GE: Fecal samples collected from genetically engineered pigs. WT: Fecal samples collected from wild type pigs. Axis Y is relative abundance of total microbiota.

**Fig 5:**
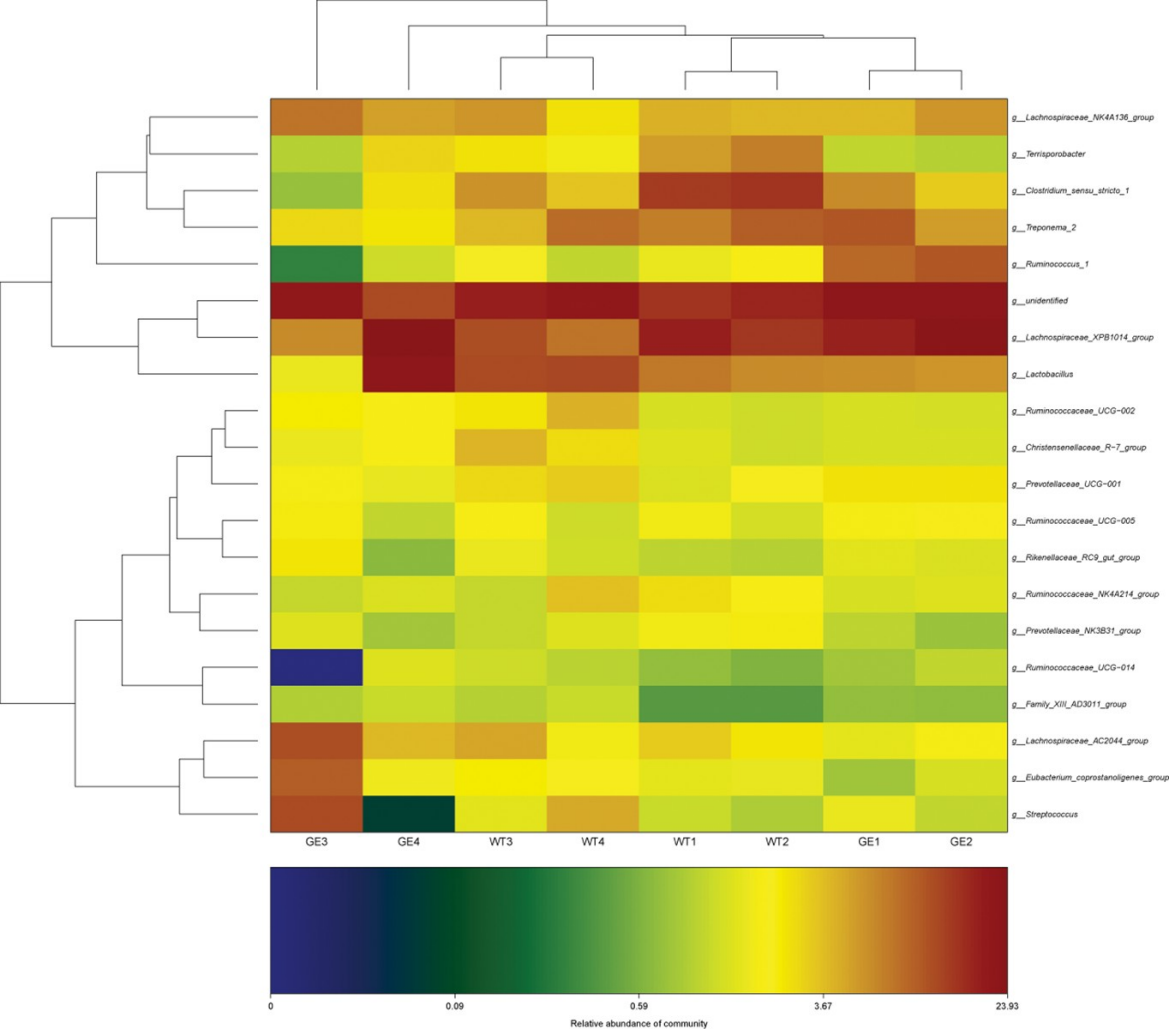
OTUs and composition of microbiota at genus level. GE: Fecal samples collected from genetically engineered pigs. WT: Fecal samples collected from wild type pigs.

**Fig 6:**
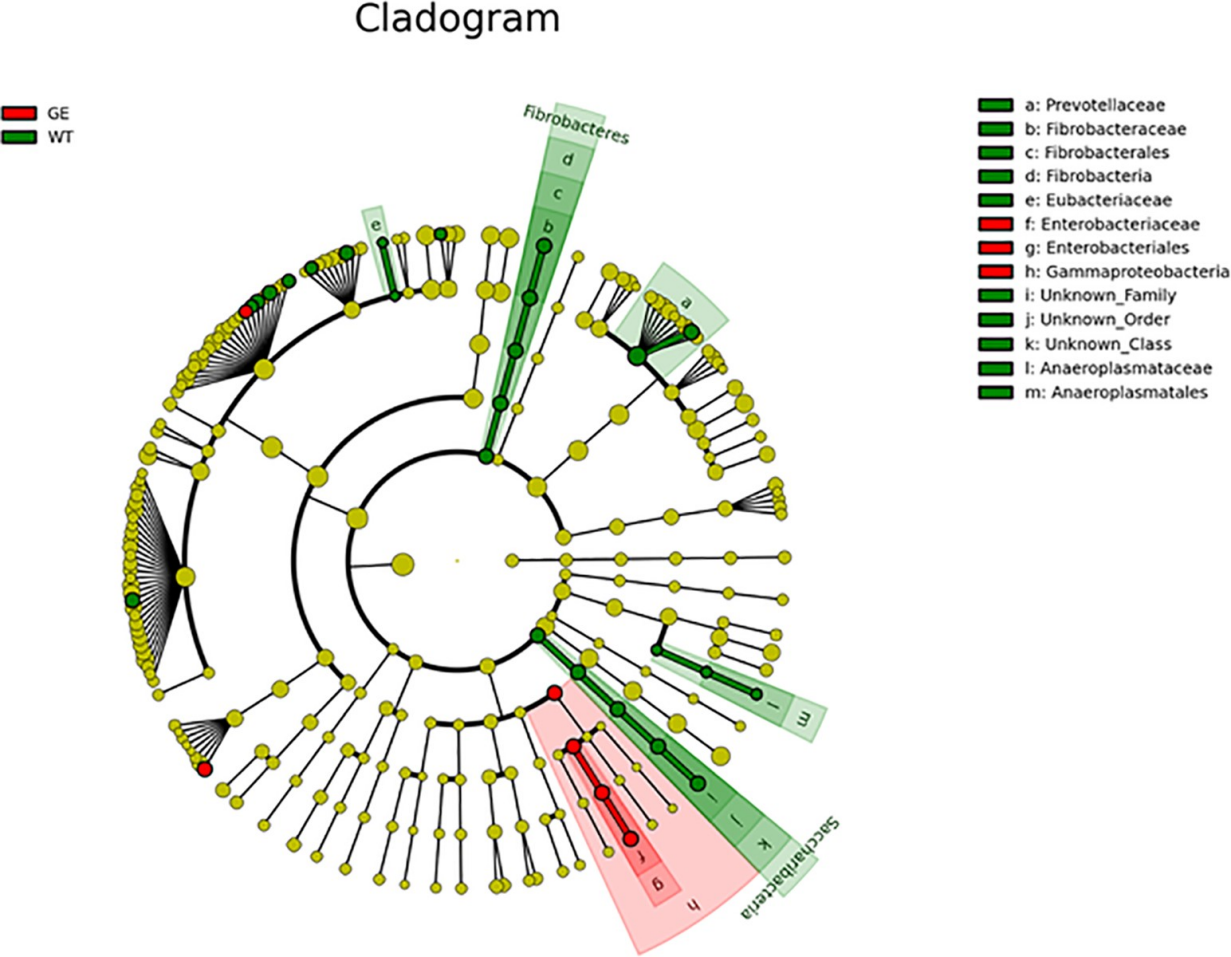
Evolutionary tree based on LEfSe analysis. GE: Fecal samples collected from genetically engineered pigs. WT: Fecal samples collected from wild type pigs. Circles from the inside to the outside represent the classification level from phylum to genera/species. Each small circle at different classification levels represents a sub-classification level, and the diameter of each small circle is proportional to the relative abundance. The yellow color indicates no significant differences between species. Red dots and green dots represent those microbial groups that play important roles in the red and green groups, respectively.

According to the results from taxonomic analysis, the composition from multiple samples were analyzed by OTU cluster analysis. As shown in **Fig 3**, at the phylum level, Firmicutes (74.7±8.6%) was the highest enriched phylotype in the *MSTN*^*-/-*^ pigs, which accounted for from 63.9% to 86.5% of relative abundance, respectively. Bacteroidetes (16.7±4.4%) accounted for from 21.0% to 10.0% of the abundance. Firmicutes and Bacteroidetes are two predominant phyla in the fecal samples from WT pigs, which showed 73.7±4.3% and 17.1±2.6% of relative abundance, respectively. Results from t-test indicated that there were not significant differences at phylum, class (**S4 Fig**) and order (**S5 Fig**) levels between *MSTN*^*-/-*^ and WT pigs. At family level, the dominant microbes were Lachnospiraceae (34.8±4.0%, 27.2±4.7%), Ruminococcaceae (17.5±2.9%, 15.2±3.0%) and Lactobacillaceae (8.9±2.9%, 9.5±2.5%) in both *MSTN*^*-/-*^ and WT pigs (see **Fig 4**). Statistical analysis showed that there was not significant difference in dominant microbes between *MSTN*^*-/-*^ and WT pigs (p values: 0.10, 0.38, 0.91), although levels of Prevotellaceae (4.6±0.86% in MSTN^-/-^; 6.3±0.84% in WT; P = 0.02), Anaeroplasmataceae (0.0015% in MSTN^-/-^; 0.0120% in WT; P = 0.02), and Eubacteriaceae (0.0000% in MSTN^-/-^; 0.0049% in WT; P = 0.04) in *MSTN*^*-/-*^ pigs were lower than in WT pigs. At genus level (**Fig 5**), Lachnospiraceae_XPB1014 group (17.5±7.0%), Lactobacillus (8.9±7.8%), Lachnospiraceae_NK4A136 group (5.6±1.4%), Treponema_2 (5.3%±3.1%), and Ruminococcus_1 (5.2±4.5%) were the top five genera in *MSTN*^*-/-*^ groups, while Lachnospiraceae_XPB1014 group (12.7±3.7%), Lactobacillus (9.5±2.5%), Clostriduim-sensu-stricto-1 (9.4±4.8%), Treponema 2 (7.3±2.1%), and Lachnospiraceae NK4A136_group (4.2±1.1%) were the most abundant genera in WT groups, but there was no significant difference in dominant strains between *MSTN*^*-/-*^ and WT pigs. LDA Effect Size analysis method was used to compare the number of bacteria at family and genus levels between *MSTN*^*-/-*^ and WT groups. As shown in **Fig 6**, Prevotellaceae (4.4%), Clostridiales (1.9%), Fibrobacteraceae (0.04%), Eubacteriaceae (0.0049%), and Anaeroplasmataceae (0.005%) in *MSTN*^*-/-*^ groups were significantly lower (p values: 0.03, 0.01, 0.01, 0.04, 0.02) than in WT groups (5.9%, 3.4%, 0.15%, 0.00%, and 0.012%, respectively), but Enterobacteriaceae (including *Escherichia coli*) (0.28%) in *MSTN*^*-/-*^ group was significantly greater than in WT group (0.026%) (p value: 0.002).

## Discussion

Up to now, intestinal microbes have been determined in a variety of animals such as chickens[15], humans[16], rodent, pigs, elephants and donkeys[17], Mosquitoes[18] and Hydra[19]. The establishment of intestinal microflora is a very complicated process that is affected by environmental factors, species, development stages, and different structure and physiological characteristics of various parts of the digestive tract. These various factors lead to the difference in the number and composition of microbial colonies in the body[20]. A relatively stable microbial ecosystem and the interactions between those intestinal microbes promote the co-evolution of complex animal symbiosis and play an important role in promoting the normal growth and development and health of animals.

We selected rectal stool for sequencing analysis because the colorectal microbes are more stable than the small intestines that are directly connected to stomach[21], and it is easier to investigate the effect of *MSTN* gene editing on intestinal microbial community structure in Meishan pigs.

Under the long-term stable environmental conditions, the fecal microbes in Meishan pigs underwent a long period of evolution and thus become relatively stable. The changes in skeletal muscle generated by ZFN editing technology in Meishan pigs may lead to changes in metabolism and thus in the diversity of fecal microbes. Many studies demonstrate that, at the phylum level, Firmicutes and Bacteriodetes are the dominant microbes in pig's rectal contents. Moreover, it was noted that Firmicutes increased significantly while Bacteriodetes decreased significantly in the obesity-type fecal microbiological composition compared to that of the lean type. The results from our current study is consistent with earlier reports[6]. In our study, no significant difference was observed in Firmicutes and Bacteriodetes between *MSTN*^*-/-*^ group and WT group. Although there were differences between the main microbial contents, the differences were not significant, indicating that although *MSTN*-editing increased the lean meat rate of Meishan pigs, it did not have a significant impact on the main microbial composition in feces.

In this study, Prevotella and Clostridium decreased in *MSTN*^*-/-*^ group and this observation is the same as reported previously[21]. This may reflect the fact that there is a relative reduction in the inflammatory response and an enhanced resistance to inflammation in *MSTN*^*-/-*^ Meishan pigs. However, Prevotella has an important role in the degradation of plant non-fibrous polysaccharides[22], and the amount of those cellulose degrading bacteria such as Eubacteriaceae and Fibrobacteraceae also significantly reduced[23]. Thus, the utilization of plant carbohydrates by GE Meishan pigs is not as effective as by WT pigs, which may be the reason why *MSTN*^*-/-*^ pigs have lower fat deposition in GE pigs. Based on the results of OTU analysis, level of Enterobacteriaceae in GE group increased. Escherichia-Shigella was the major bacteria of fecal *Escherichia coli*. Escherichia-Shigella is not pathogenic and is found in human and animal intestinal tract, has no effect on the health of the body. However, the secretion of lipopolysaccharide endotoxin by Escherichia-Shigella may cause obesity and insulin resistance in mice[24]. Along this line it is noted that our recent studies demonstrated that *MSTN*^*-/-*^ Meishan pigs produce pork with greater percentage of lean meat with lower level of fat[6], with the insulin sensitivity being significantly increased in these MSTN^-/-^ Meishan pigs when compared to WT pigs[25]. This clearly indicated that the increase in Enterobacteriaceae in MSTN^-/-^ Meishan pigs did not cause obesity or a decrease in insulin sensitivity.

After α test of samples in two groups, it was concluded that microbial diversity in *MSTN*^*-/-*^ Meishan pigs is slightly lower than in WT Meishan pigs, but the difference is not significant. This indicates that gene editing technology did not have significant effect on gut microbiota in *MSTN*^*-/-*^ Meishan pips.

## Conclusion

From the analysis of 16S sequencing data, the investigated bacterial community was mostly stable between MSTN^-/-^ and WT pigs, yet, certain bacterial groups were selectively promoted, but they don’t have any adverse effect on pigs’ healthy status. Therefore, our current study suggests that ZFN mediated MSTN loss-of-function mutation (*MSTN*^*-/-*^*)* did not adversely affect the compositional structure of fecal microbiota in Meishan pigs.

## Supporting information

S1 Fig Multiple samples rarefaction curves. Fecal samples collected from genetically engineered pigs. WT: Fecal samples collected from wild type pigs. Axis x: random sequencing data. Axis Y: observed OTUs.
(TIF)

S2 Fig Multiple samples Shannon-Wiener curves. GE: Fecal samples collected from genetically engineered pigs. WT: Fecal samples collected from wild type pigs. Axis x: Shannon index, axis Y: number of sequencing
(TIF)

S3 Fig Multiple samples species accumulation curves. GE: Fecal samples collected from genetically engineered pigs. WT: Fecal samples collected from wild type pigs. Axis x sample size, axis Y: OTU number.
(TIF)

S4 Fig Microbial community structure of fecal samples at class level. GE: Fecal samples collected from genetically engineered pigs. WT: Fecal samples collected from wild type pigs. Axis Y is relative abundance of total microbiota.
(TIF)

S5 Fig Microbial community structure of fecal samples at order level. GE: Fecal samples collected from genetically engineered pigs. WT: Fecal samples collected from wild type pigs. Axis Y is relative abundance of total microbiota.
(TIF)

S1 Table Contents of nutrients in diets feeding Meihan pigs. (DOCX)

S2 Table α-diversity index in GE and WT. GE: Fecal samples collected from genetically engineered pigs. WT: Fecal samples collected from wild type pigs.
(DOCX)
