# Supplementary figures and images for: Effect of ZFN-edited myostatin loss-of-function mutation on gut microbiota in Meishan pigs
Source: PLoS One. 2019 Jan 15;14(1):e0210619. doi: 10.1371/journal.pone.0210619 (PMC6333347; doi:10.1371/journal.pone.0210619)

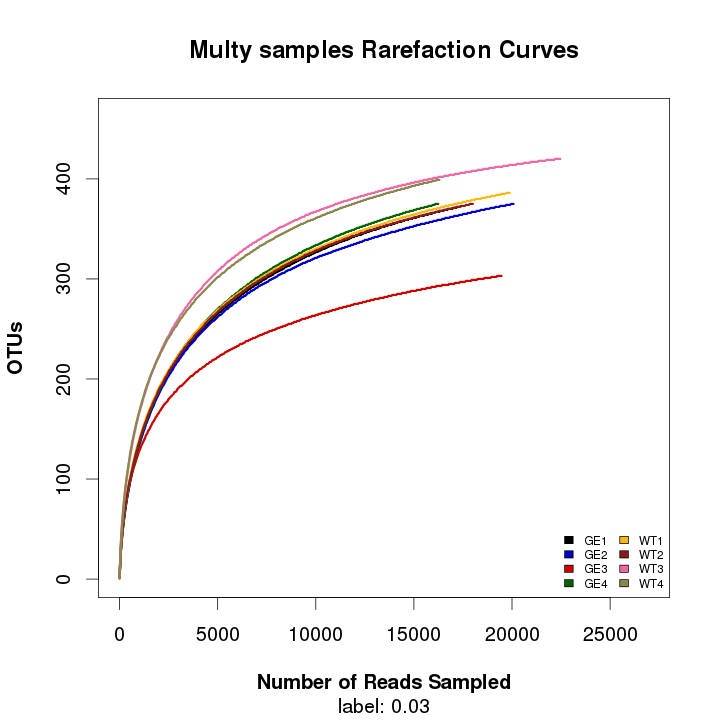

Supplement: S1 Fig — Fecal samples collected from genetically engineered pigs. WT: Fecal samples collected from wild type pigs. Axis x: random sequencing data. Axis Y: observed OTUs. (TIF) [file pone.0210619.s001.tif]

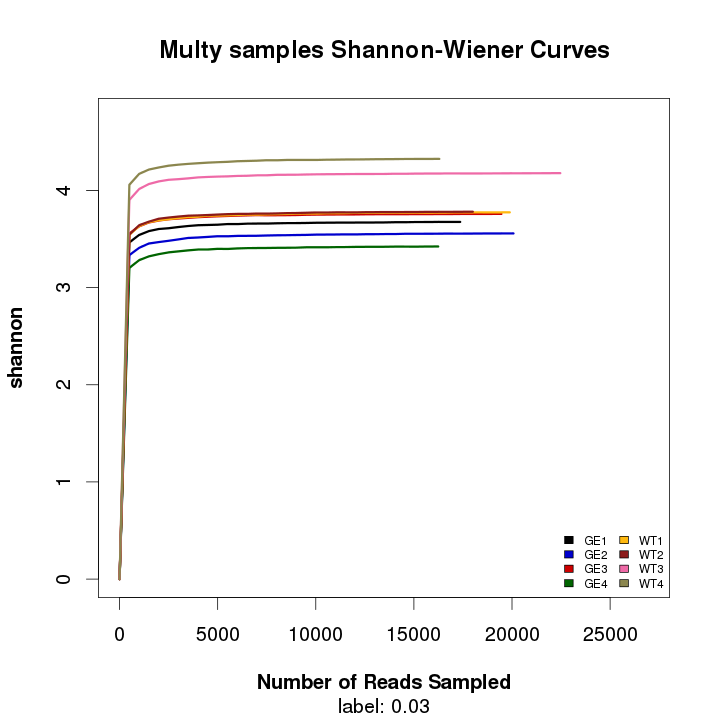

Supplement: S2 Fig — GE: Fecal samples collected from genetically engineered pigs. WT: Fecal samples collected from wild type pigs. Axis x: Shannon index, axis Y: number of sequencing (TIF) [file pone.0210619.s002.tif]

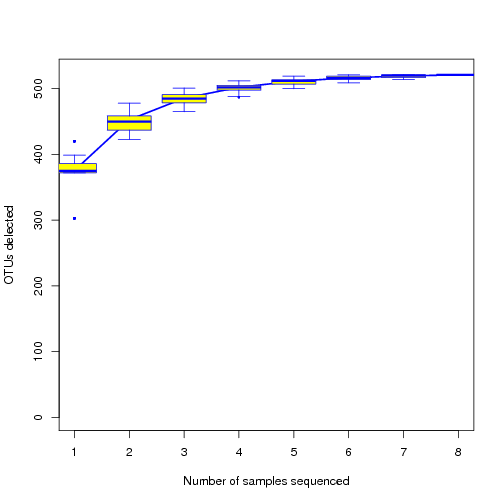

Supplement: S3 Fig — GE: Fecal samples collected from genetically engineered pigs. WT: Fecal samples collected from wild type pigs. Axis x sample size, axis Y: OTU number. (TIF) [file pone.0210619.s003.tif]

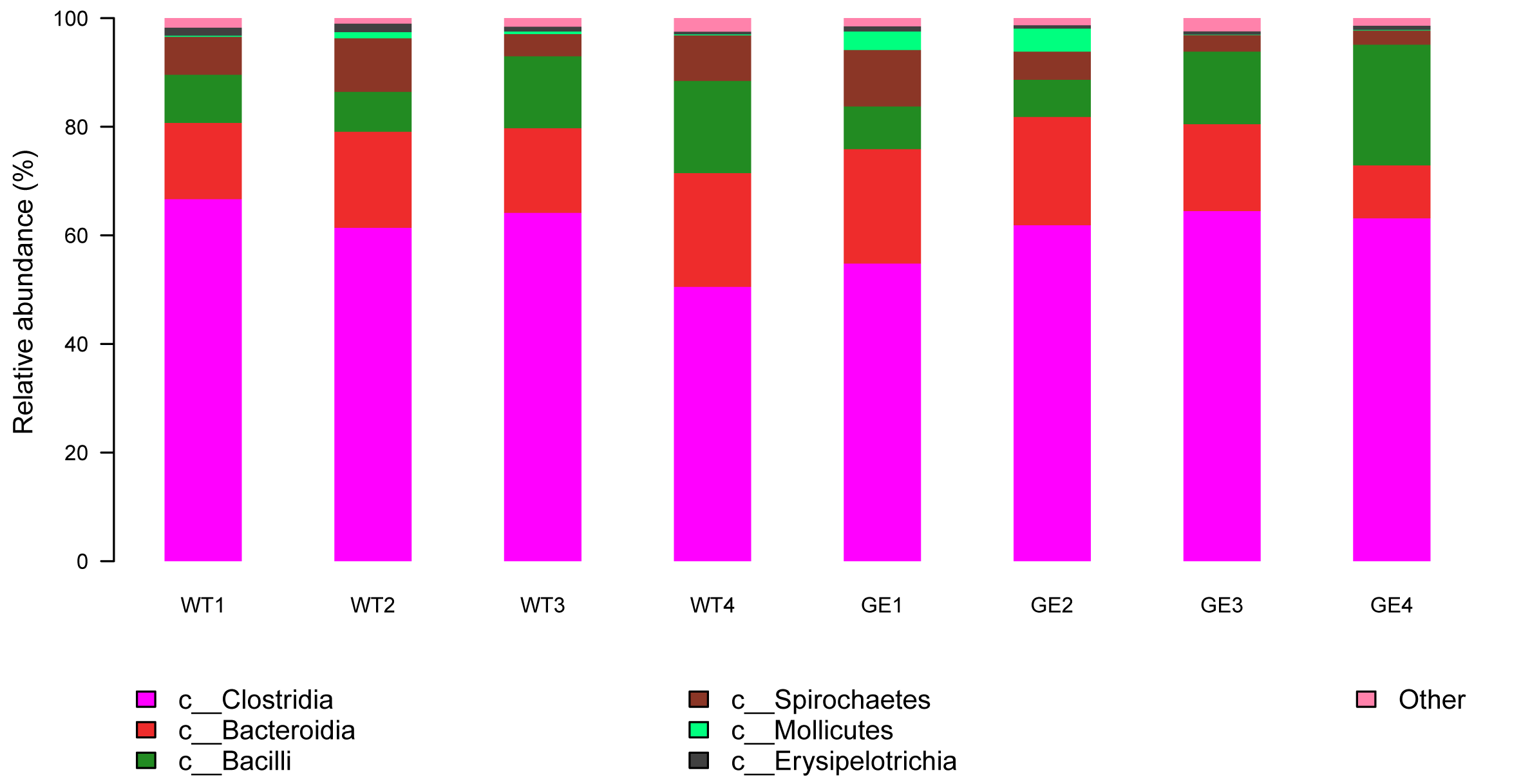

Supplement: S4 Fig — GE: Fecal samples collected from genetically engineered pigs. WT: Fecal samples collected from wild type pigs. Axis Y is relative abundance of total microbiota. (TIF) [file pone.0210619.s004.tif]

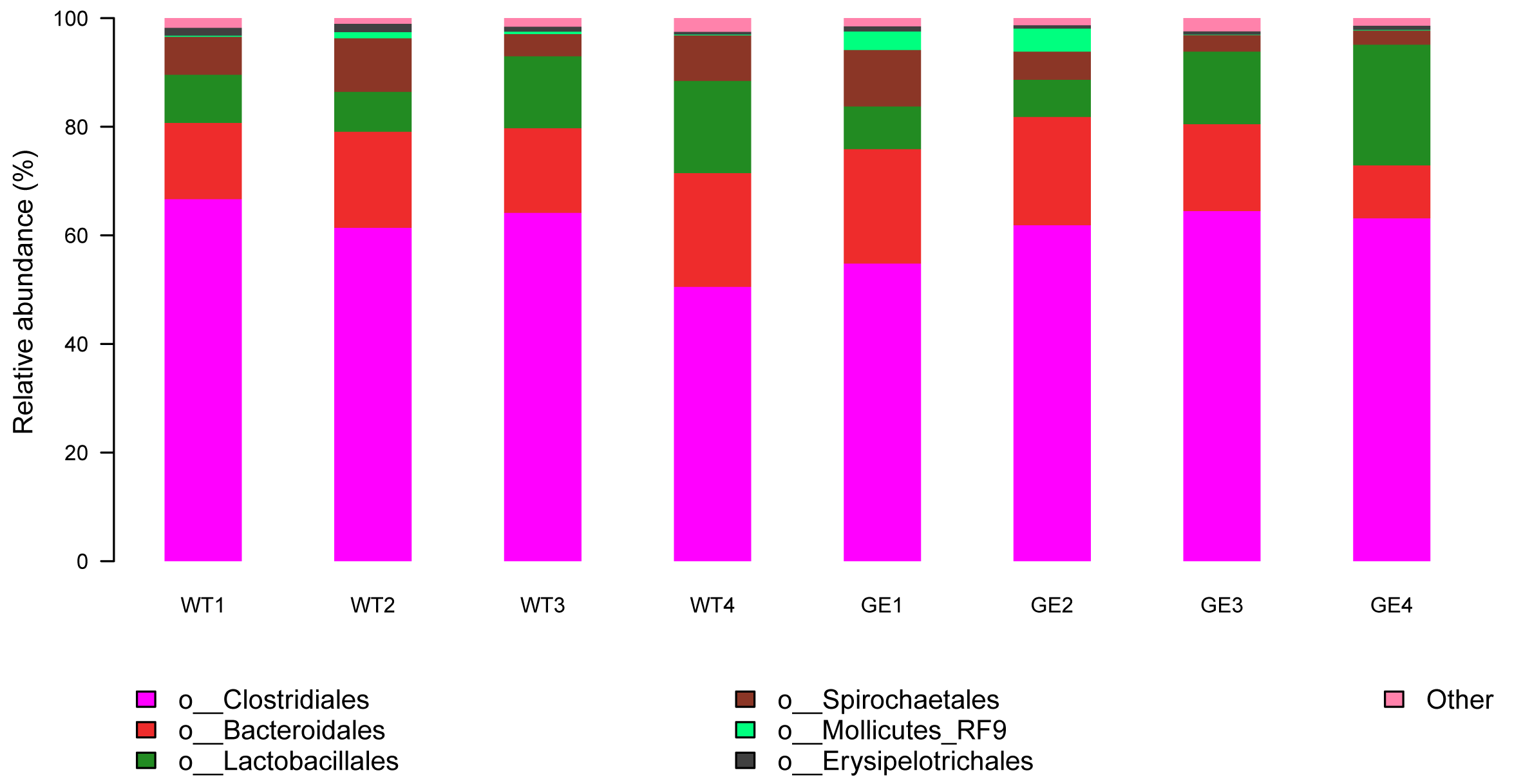

Supplement: S5 Fig — GE: Fecal samples collected from genetically engineered pigs. WT: Fecal samples collected from wild type pigs. Axis Y is relative abundance of total microbiota. (TIF) [file pone.0210619.s005.tif]
