# Supplementary material for: Effect of ZFN-edited myostatin loss-of-function mutation on gut microbiota in Meishan pigs
Source: PLoS One. 2019 Jan 15;14(1):e0210619. doi: 10.1371/journal.pone.0210619 (PMC6333347; doi:10.1371/journal.pone.0210619)
Supplement: S1 Table — (DOCX) [file pone.0210619.s006.docx]

**S1 Table** Contents of nutrients in diets feeding Meihan pigs

| Name of diets | Feed at (body weight) | CP (%)≥ | CF (%)≤ | Ash (%)≤ | Ca (%) | P (%)≥ | NaCl (%) | Lys (%)≥ |
| --- | --- | --- | --- | --- | --- | --- | --- | --- |
| Dai Ru Bao | <4kg | 21.00 | 4.00 | 7.00 | 0.7-1 | 0.55 | 0.3-1.0 | 1.45 |
| Ru Zhu Bao | 4-16kg | 20.00 | 5.00 | 7.00 | 0.6-0.9 | 0.50 | 0.3-1.0 | 1.30 |
| Zi Zhu Bao | 16-32kg | 19.00 | 5.00 | 7.00 | 0.6-0.9 | 0.50 | 0.3-1.0 | 1.15 |
| 551 | 32-45kg | 18.00 | 5.00 | 7.00 | 0.6-0.9 | 0.45 | 0.3-0.8 | 1.10 |
| 552 | 45-60kg | 15.00 | 9.00 | 7.00 | 0.5-1.4 | 0.40 | 0.3-0.8 | 0.80 |
| 553 | 60-100kg | 17.00 | 7.00 | 8.00 | 0.5-1.4 | 0.45 | 0.3-0.8 | 0.95 |

CP: crude protein; CF: crude fiber; Ash: crude ash
