# Supplementary material for: Effect of ZFN-edited myostatin loss-of-function mutation on gut microbiota in Meishan pigs
Source: PLoS One. 2019 Jan 15;14(1):e0210619. doi: 10.1371/journal.pone.0210619 (PMC6333347; doi:10.1371/journal.pone.0210619)
Supplement: S2 Table — GE: Fecal samples collected from genetically engineered pigs. WT: Fecal samples collected from wild type pigs. (DOCX) [file pone.0210619.s007.docx]

**S2 Table** α-diversity index in GE and WT

| Sample ID | Chao1 | Goods_ coverage | Observed _species | PD_ whole_ tree | shannon |
| --- | --- | --- | --- | --- | --- |
| GE1 | 407.92 | 0.99 | 357.90 | 31.42 | 5.30 |
| GE2 | 416.77 | 0.99 | 351.00 | 31.53 | 5.13 |
| GE3 | 339.77 | 1.00 | 287.50 | 27.28 | 5.42 |
| GE4 | 432.75 | 0.99 | 367.30 | 32.44 | 5.44 |
| WT1 | 423.14 | 0.99 | 361.00 | 31.84 | 5.44 |
| WT2 | 422.32 | 0.99 | 360.90 | 32.13 | 5.45 |
| WT3 | 438.27 | 1.00 | 392.60 | 33.88 | 6.02 |
| WT4 | 441.54 | 0.99 | 390.50 | 34.30 | 6.24 |
| P-value | 0.18 | 0.90 | 0.13 | 0.12 | 0.07 |

GE: Fecal samples collected from genetically engineered pigs. WT: Fecal samples collected from wild type pigs.
